# Research on urban path selection of construction vehicles based on bi-objective optimization

**DOI:** 10.1371/journal.pone.0275678

**Published:** 2022-10-06

**Authors:** Mengkai Liu, Zepeng Xu

**Affiliations:** Evergrande School of Management, Wuhan University of Science and Technology, Wuhan, Hubei, China; University of Alicante, SPAIN

## Abstract

With the implementation of urban central rail transit and old city reconstruction projects, construction vehicles frequently enter and depart the urban area. And because of its large volume and other characteristics, it increases the risk probability and severity of urban traffic accidents. This study takes the transportation path selection of construction vehicles as the breakthrough point, weighs the transportation efficiency and safety of construction vehicles, establishes a bi-objective optimization model, involving constraints such as height limit, weight limit, speed limit, direction limit and traffic limit and uses genetic algorithm to solve it. Finally, through case analysis, the user preference is adjusted to conduct functional test and description of the model. The results indicate that the model has the function of transportation vehicle path optimization. In the meantime, compared with the safest route, the time-consuming of the optimal route decreases by 16% and the risk increases by 7.4%, while the time-consuming of it increases by 5% and the risk decreases by 15.4% compared with the shortest route. Moreover, the corresponding coefficients of time-consuming and safety preference can reach about 0.65, and the relevant stakeholders have high acceptance of the route. Further improvement of construction vehicle management mechanism based on path optimization is one of the limited ways to effectively improve the current situation of construction vehicle management.

## 1. Introduction

With the rapid development of global urbanization and the increasing number of cars, traffic safety has become a worldwide problem, and road transportation is the most prone to traffic accidents [1]. Among them, heavy goods vehicles (HGV) represented by construction vehicles account for a large proportion of accidents. In 2006, there were 3114 fatal HGV accidents in 15 EU countries, accounting for about 13% of total road deaths [2]. More than half of the bike accidents, particularly in London, are linked to construction vehicles, which cause more damage than other vehicles due to their large volume [3, 4]. In China, with the rapid development of urbanization, urban construction continues to accelerate, old city renovation, rail transit and other construction projects continue to increase, resulting in a variety of construction materials, construction waste and other transportation needs are also growing [5]. And for a high-rise building with a total commercial concrete volume of about 10000 m^3^, G4805E No.5 concrete mixing vehicle is required to travel to and from the mixing station-project 1700 shifts. It can be seen that construction vehicles frequently enter and leave the urban area, which increases the traffic flow. It also becomes one of the important sources of traffic accident risk due to complex urban road conditions and unfamiliarity of drivers with urban roads.

There are many reasons for the frequent accidents of construction vehicles. On the one hand, construction vehicles are large in volume, wide in blind area and difficult in vehicle control technology. On the other hand, many drivers have bad living habits such as drug abuse or alcohol abuse, poor diet and sleep quality, and lack of exercise [6–8]. In the meantime, long working hours and poor working environment jointly cause driving HGV to become one of the highest mortality occupations [9, 10]. Moreover, poor road infrastructure, poor law enforcement by government departments, and insufficient training of drivers by transportation enterprises are also important incentives for road accident risks [11]. Therefore, it is necessary to study the urban driving of construction vehicles to improve the efficiency and safety of vehicle transportation in order to ensure the common interests of transportation enterprises and the public.

In the study of construction vehicle transportation, innovative models of third-party safety supervision institutions or platforms have received wide attention from government and academia. Jiao et al. constructed an evolutionary game model of government safety supervision and road transportation enterprises ’ safety production, and believed that improving the supervision ability of third-party safety supervision institutions was of great significance to promote road transportation enterprises to choose safety production strategies [12]. Traffic monitoring system based on machine learning is used to monitor urban traffic congestion by identifying heavy construction vehicles on urban traffic congestion sections [13]. Positioning system is also widely used in vehicle scheduling management to achieve real-time monitoring of construction vehicles [14, 15]. Liang et al. studied the influence of drivers ’ EEG and dermal electric activities on risk perception [16]. Guest et al. studied the relationship between driver age and accident probability [17]. It can be seen that the management and scheduling of construction vehicles and driving safety are the hot topics of scholars, mainly focusing on vehicle monitoring, driving technology assistance and drivers themselves. However, there is no systematic analysis on the transportation route of construction vehicles. While transportation route planning is widely used in transportation problems such as cold chain logistics, emergency commodities and refined oil [18–20]. At the same time, when choosing routes, drivers are mainly based on their own experience or navigation software. Different drivers have different choices, and they are unknown about transportation time and safety [21]. Moreover, the existing navigation software mainly considers the timeliness of routes, and then selects the shortest transportation time route, which fails to effectively deal with the relationship between vehicle transportation efficiency and safety, and has poor applicability to the field of construction vehicle transportation [22, 23]. Therefore, it is necessary to consider the urban driving characteristics of construction vehicles, weigh their transportation efficiency and safety, and optimize and clarify the urban driving route of construction vehicles.

Early path optimization is mainly aimed at single-objective problems, usually with the shortest transportation distance or time as the goal [24–26]. However, due to the difficulty in reflecting the trade-off between efficiency and safety of users in studying single-objective problems, scholars have launched research on multi-objective path optimization problems, and such problems have more research results [27–29]. Different from single-objective optimization, the sub-objectives in multi-objective optimization may conflict with each other. The improvement of one sub-objective may reduce the performance of the other sub-objective, and it is difficult to make multiple sub-objectives achieve optimal simultaneously. To solve this problem, some scholars designed scenario-based algorithms to obtain Pareto optimal solution, which was to improve the optimal solution of sub-objective B without reducing the performance of sub-objective A [30, 31]. Or according to the importance of different objectives, the corresponding weight coefficient was given or the weighted-sum method was used to transform the multi-objective problem into a single-objective problem [32–35]. In order to fully reflect the influence of user preference on path selection, this study obtains the optimal route by adjusting the weight coefficient of sub-goals and reveals the response law of preference and path selection.

Genetic algorithm has strong global search ability and effectively avoids falling into local optimum, so it is widely used in multi-objective path selection problem. Ma et al. set up taxi optimization model based on improved multi-objective genetic algorithm to increase driver income and reduce passenger travel cost [36]. Xue et al. used non-dominated sorting genetic algorithm (NSGA-II) to select shorter and safer routes for mobile robots [37]. Ben Alaia et al. found the shortest distance, the shortest delay time and the least vehicle route by improving the coding chromosome representation path method in genetic algorithm [38]. Therefore, this study, combined with the actual situation of urban driving construction vehicles, selects genetic algorithm as a multi-objective optimization method.

Relevant literature indicated that urban road risk sources mainly include population quantities, the number of population-intensive points such as schools, the number of bridges and turns, and whether there is non-motorized lane on both sides of the road, as shown in Table 1.

**Table 1:** Summary of urban road risk sources.

Researchers	Research content	Risk sources
Batta et al.; Pradhananga et al.	A population exposure model was established to measure road risks using the total population during transportation [39, 40].	Population quantities
Bronfman et al.; Wang et al.	The concept of vulnerable centers was introduced into the hazardous material routing problem, and the distribution route far from vulnerable centers was selected [41, 42].	Number of densely populated points such as schools
Huang et al.	Compared with ordinary roads, bridge traffic accidents are often more serious, which often lead to secondary accidents and long-term traffic congestions. And because of their large load, construction vehicles will cause a certain degree of hidden damage to the bridges [43].	Number of bridges
Zhang et al.; Dooley et al.	In view of the traffic safety problems caused by semi-trailer turn inner wheel difference and visual blind area, the obstacle hazard identification trajectory model in turning blind area was established to reduce the probability of traffic accidents caused by turning blind area [44, 45].	Whether to turn
Zhang et al.	The optimization design of non-motorized lane was proposed. The hard isolation measures were used to divide the fast and slow traffic and avoid taking up the motorized lane such as take-out and express delivery, which were conducive to improving the road operation efficiency and safety level [46].	Non-motorized lane on both sides of the road

Therefore, based on the characteristics of construction vehicles and their driving effects in urban areas, this study summarizes the sources of urban road risk, and establishes a path optimization model with the shortest transportation time and the minimum risk as the bi-objective. Through the simulation analysis of actual cases, the validity and feasibility of the method are demonstrated, and the construction vehicle management mechanism based on path optimization is proposed, which provides strong support for effectively protecting the benefits and safety of transportation enterprises and urban residents.

## 2. Model construction

For the construction vehicle urban transportation problem, the timeliness of the construction vehicle driving route has important value to the construction unit project progress, cost, etc. In the meantime, urban residents also have high requirements for vehicle safety driving. Therefore, this study selects the shortest construction vehicle transportation time and minimum risk as the bi-objective.

### 2.1 Symbol description

The main variable symbol definitions involved in the model are shown in Table 2.

**Table 2:** Symbol description.

Symbols	Definition
*A*	Road Traffic Network
*i*,*j*,*k*	Node number of Road Network *A*
(*i*,*j*)	The road between two nodes *i*,*j*
*x*_*ij*_	Whether road (*i*,*j*) passes or not
*d*_*ij*_	The road distance between two nodes *i*,*j*
*p*_*ij*_	Number of traffic lights between road (*i*,*j*)
*β*_*ij*_	Road condition complexity of road (*i*,*j*), *β*_*ij*_ = e^-p^*^q^, *q* is a constant coefficient
*γ*_*ij*_	The complexity of traffic flow on road (*i*,*j*), determined by the single-day traffic flow, can be classified as very smooth, smooth, lightly congested, moderately congested, and severely congested [47]
*α*_*ij*_	Road (*i*,*j*) resistance coefficient of traffic, *α*_*ij*_ = *β*_ij_*γ_*ij*_
*V*_*ij*_	Design speed limit of road (*i*,*j*)
*v*_*ij*_	The actual average travel speed of road (*i*,*j*), *v*_*ij*_ = *V*_ij_*α_*ij*_
*ρ*_*ij*_	Population density on both sides of road (*i*,*j*)
*num*_*ij*_	Number of schools, hospitals, shopping malls on both sides of the road (*i*,*j*)
*bri*_*ij*_	Whether the road (*i*,*j*) passes through the bridge
*turn*_*ij*_	Whether the road (*i*,*j*) turns
*non*_*ij*_	Whether the road (*i*,*j*) has a non-motorized lane
*h*	Body height of construction vehicles
*g*	Maximum load of construction vehicles
*H*_*ij*_	Road (*i*,*j*) height limit
*G*_*ij*_	Road (*i*,*j*) weight limit

### 2.2 Objective function

The model takes the shortest transportation time and the smallest risk as the bi-objective.

Transportation time is mainly affected by the road distance and the actual speed, where the actual speed is affected by the road conditions and the complexity of traffic flow. Therefore, the shortest time-consuming function is established and expressed in the form shown in Eq (1):

$$f_{1}=min\sum_{(i,j)\in A}d_{ij}/v_{ij}$$ (1)

Based on the summary of risk sources in Table 1, the risk minimization function is established and expressed in the form shown in Eq (2):

$$f_{2}=min\sum_{(i,j)\in A}\rho_{ij}e^{a*num_{ij}+b*bri_{ij}+c*turn_{ij}+d*non_{ij}}$$ (2)

where *a*, *b*, *c*, and *d* are constant coefficients.

Establish the integrated objective function *F* as in Eq (3):

$$F=100w_{1}\frac{f_{1}}{f_{1}^{*}}+100(1‐w_{1})\frac{f_{2}}{f_{2}^{*}}$$ (3)

where *w*_1_ is the time-consuming preference coefficient, taking values from 0 to 1, then 1-*w*_1_ is the safety preference coefficient. And *f*_1_* and *f*_2_* are the integrated objective function values when *w*_1_ = 1 and *w*_1_ = 0, respectively, representing only the shortest time-consuming or risk minimization is considered.

### 2.3 Constraint conditions

**Fig 1:**
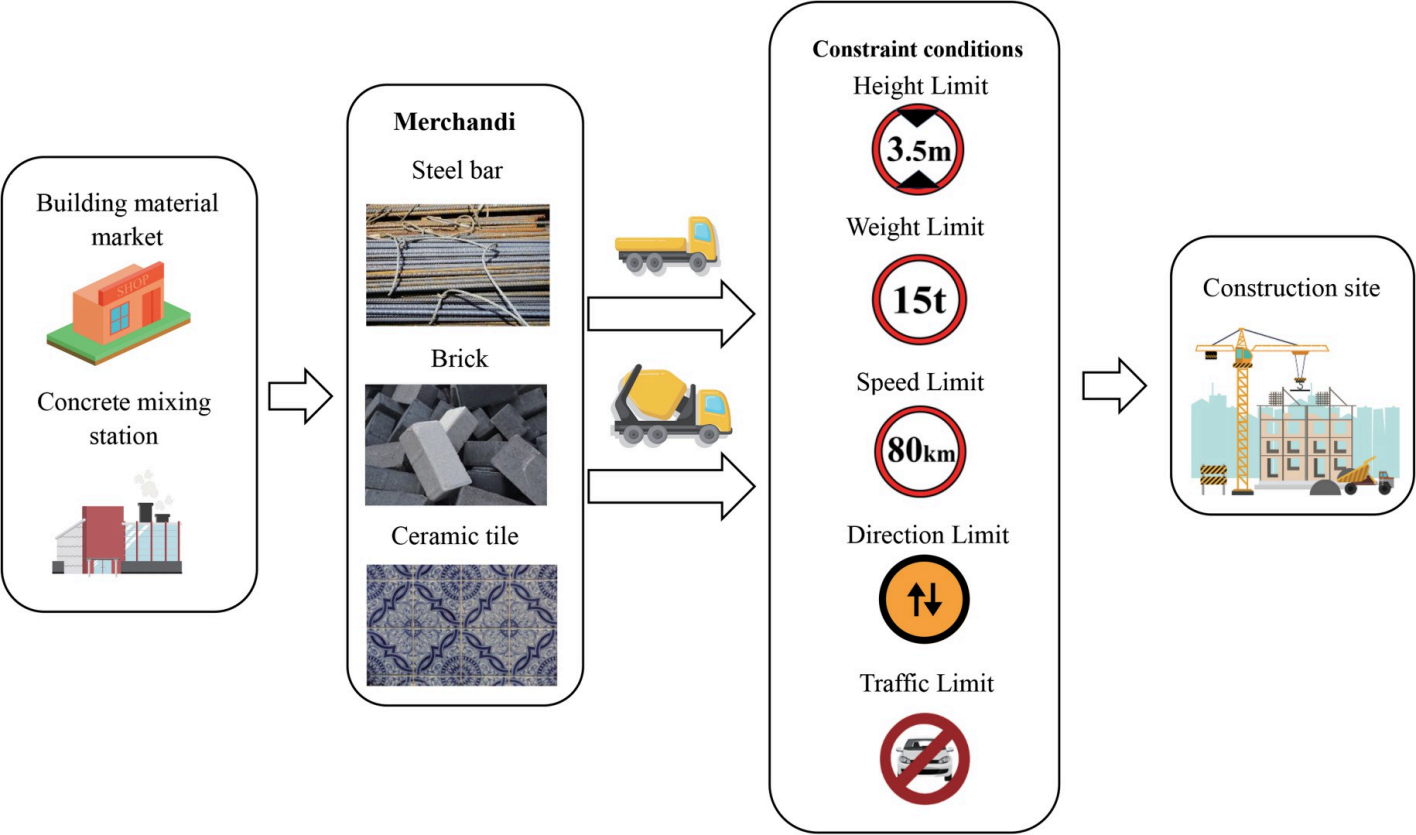
Constraint conditions.

Compared with other transport vehicles, construction vehicles have the characteristics of large volume and heavy load. Therefore, as shown in Fig 1, when selecting paths, drivers need to consider the limit conditions of height, weight and speed of roads or bridges.

Road height limit constraint:

$$\forall (i,j)\in A,h\leq H_{ij}$$ (4)

Bridge weight limit constraint:

$$\forall (i,j)\in A,t\leq T_{ij}$$ (5)

Road speed limit constraint:

$$\forall (i,j)\in A,v_{ij}\leq V_{ij}$$ (6)

Road direction limit constraint:

$$\exists (i,j)\in A,x_{ij}=1,x_{ji}=0$$ (7)

Road traffic limit constraint:

$$\exists (i,j)\in A,x_{ij}=x_{ji}=0$$ (8)

## 3. Model solving

### 3.1 Vehicle turn recognition

**Fig 2:**
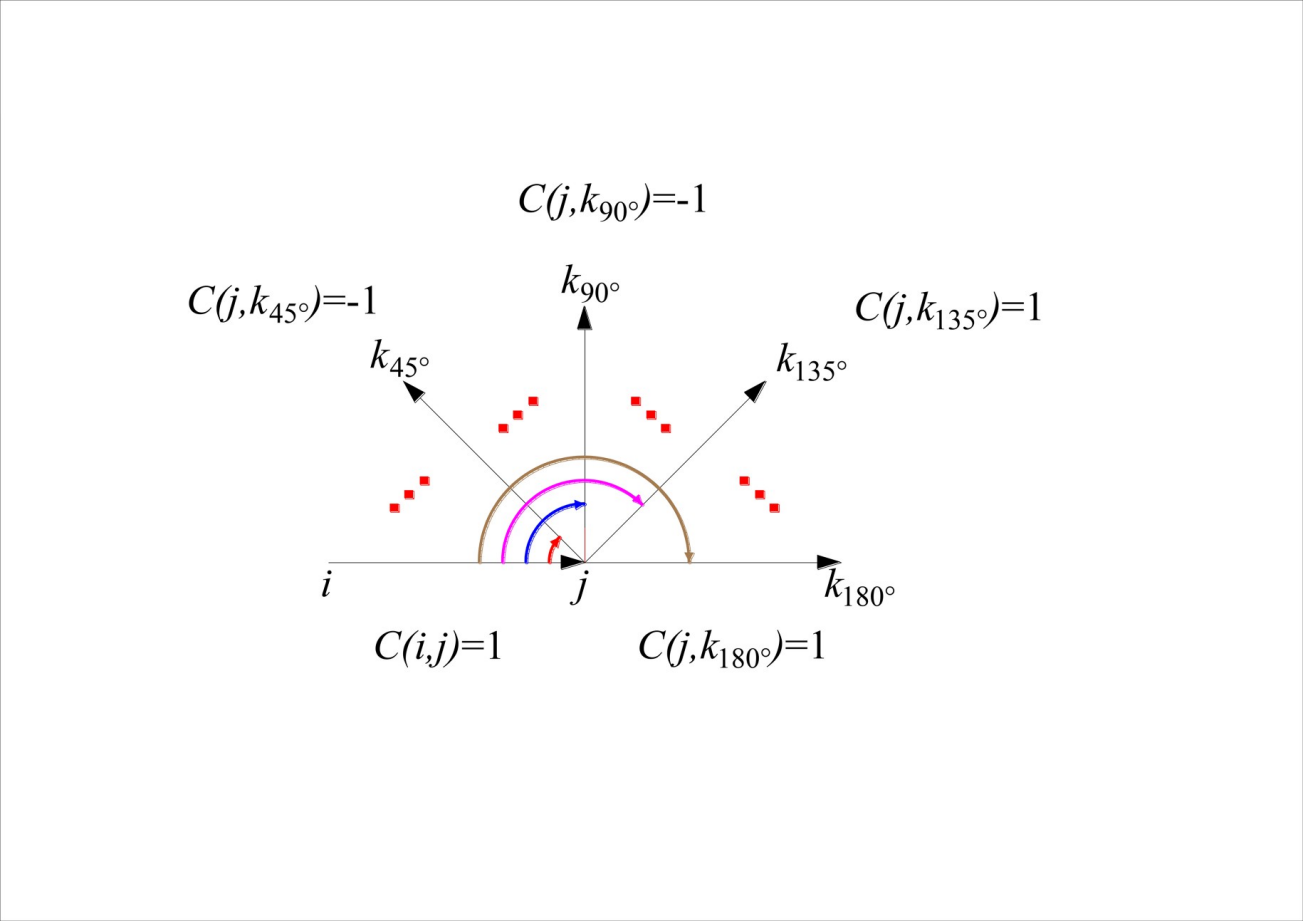
Simulated path.

The model assigns the turn attribute of the east-west road to *C*(*i*,*j*) = 1, and the turn attribute of the road with the angle less than or equal to 90° to *C*(*j*,*k*) = -1, as shown in Fig 2.

Then the turning attribute of road (*j*,*k*_*r*_) is assigned as shown in Eq (9), where *r* is the angle between road (*i*,*j*) and (*j*,*k*_*r*_) at node *j*.


$$C(j,k_{r})=\{\begin{array}{c} ‐1,0^{\circ }\leq r\leq 90^{\circ }\\ 1,90^{\circ }<r\leq 180^{\circ } \end{array}$$ (9)

Then the turn property of node *j* is shown in Eq (10):

$$C(i,j)*C(j,k_{r})=\{\begin{array}{c} 1,j\;\mathrm{point}\;\mathrm{straight}\\ ‐1,j\;\mathrm{point}\;\mathrm{turn} \end{array}$$ (10)

### 3.2 Algorithm design

**Fig 3:**
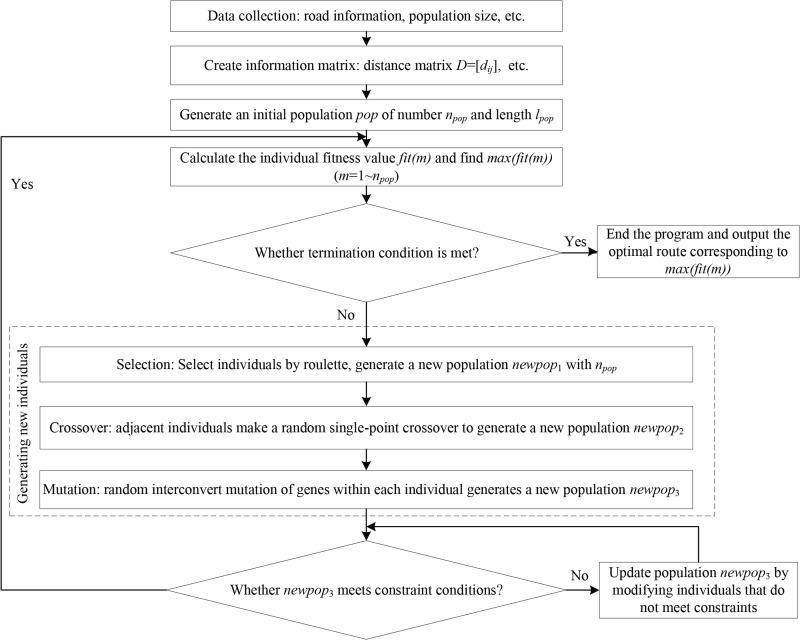
Solving steps.

In this study, genetic algorithm is used to solve the problem, and the solving steps are shown in Fig 3.

#### 3.2.1 Collect data to build information matrix

The actual map is simplified into a square network with intersections as nodes, and there are *n* nodes in the whole area from the transportation starting point to the end point. The data such as road information, population quantities, and the number of schools, hospitals and shopping malls are collected to build the information matrix, including the distance matrix *D* = [*d*_*ij*_], the population density matrix *P* = [*ρ*_*ij*_], and the actual speed matrix *V* = [*v*_*ij*_]. Taking the distance matrix *D* as an example, the matrix form is shown in Eq (11):

$$D=[\begin{array}{cccc} 0 & d_{12} & \cdots & d_{1n}\\ d_{21} & \ddots & \ddots & d_{2n}\\ \vdots & \ddots & \ddots & \vdots \\ d_{n1} & d_{n2} & \cdots & 0 \end{array}]$$ (11)

#### 3.2.2 Individual coding design

For the characteristics of the route preference problem, this study uses a random coding of natural numbers between [1-*n*], each individual corresponds to a transport route. In the meantime, the starting point 1 and the end point *n* remain unchanged. Moreover, the other natural numbers in the individual are the nodes through which the route passes.

#### 3.2.3 Establishment of fitness function

Since this study is to choose the route with the minimum value of the comprehensive objective function, considering the simple application principle of establishing the fitness function, the fitness function takes the reciprocal of the objective function:

$$fit(m)=\frac{1}{F(m)}$$ (12)

where *fit*(*m*) is the *m*th individual fitness value, *m* = 1~*n*_*pop*_.

#### 3.2.4 Operations of selection, crossover, mutation

The probability of individual being selected under the principle of roulette is:

$$pro(m)=\frac{fit(m)}{\sum_{m=1}^{n_{pop}}fit(m)}$$ (13)

where *pro*(*m*) is the probability that the *m*th individual is selected.

When *rand*<*pc*, adjacent individuals make a single-point crossover, and the crossover position is:

$$site=Round(rand*l_{pop})$$ (14)

When *rand*<*pm*, the genes within a single individual undergo interconvert mutation, and the mutation position is:

$$site_{u}=Round(rand*l_{pop})$$ (15)

where *rand* is the randomly generated number from 0 to 1, *pc* and *pm* are the probabilities of crossover and mutation, respectively, *site* is the single-point crossover location, *site*_*u*_ is the interconvert mutation location, *u* = 1,2. And *Round* is the rounding function.

#### 3.2.5 Constraint conditions

When the construction vehicle passes through the area of height limit, weight limit, speed limit, direction limit and traffic limit, it checks whether the constraints are satisfied. And if not, it needs to modify the genetic segment and generate new individuals until the constraints are satisfied.

## 4.Case study

### 4.1 Case background

**Fig 4:**
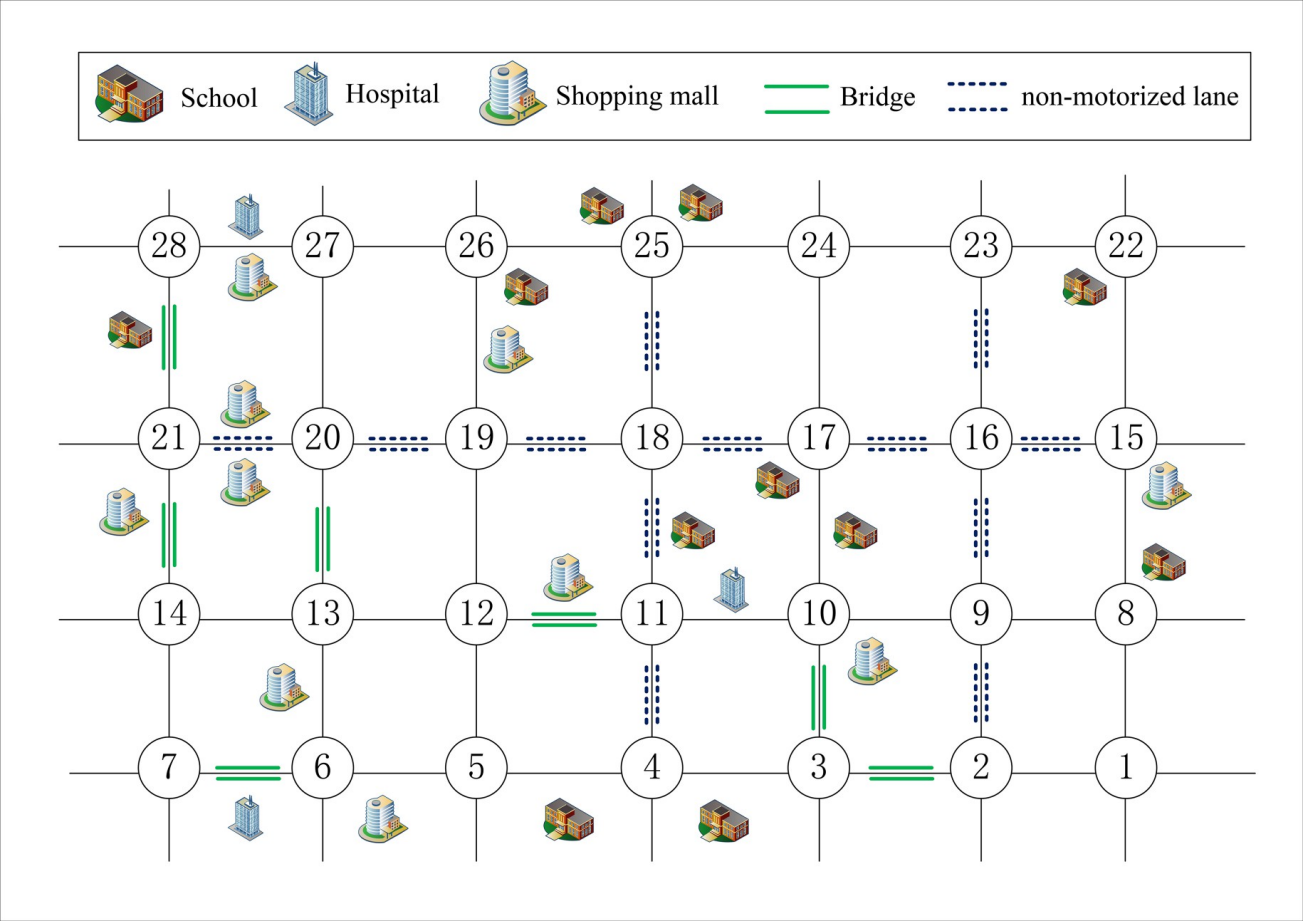
Case road network scope.

In this study, based on the case of literature [48], the scope of the road network is determined as shown in Fig 4. The area is located near the People’s Government of Haining City, with dense population, high traffic flow, and many places of science, education, culture and health. The area is about 15 km^2^, and the road network density is about 2.73 km/ km^2^, including 11 schools, 3 hospitals, 10 large and medium-sized shopping malls, 7 cross-river bridges, 57 residential communities, and the resident population is about 184,000 (shown in S1 Dataset). It is assumed that construction vehicles frequently transport building materials from position 1 to position 28. The size of the vehicle is 7 * 2.5 * 3.5m and the maximum load is 12t. And the road constraint conditions are shown in Table 3.

**Table 3:** Road constraint conditions.

Constraint conditions	Constraint sections
Height Limit	There is a pedestrian bridge with a height limit of 3m in Road (1,8), while other roads have no height limit.
Weight Limit	Maximum load of Bridge (14,21) and Bridge (21,28) is 10t, while other bridges maximum load is 15t.
Speed Limit	The Road (1,2), (2,3), (3,4), (4,5), (5,6), (6,7), (6,13), (13,20) and (20,27) speed limit is 60km/h, while speed limit of other roads is 50km/h.
Direction Limit	There is no direction limit road.
Traffic Limit	Road (23,24) is a historical and cultural block, and motor vehicles are prohibited from driving.

### 4.2 Timeliness test

**Fig 5:**
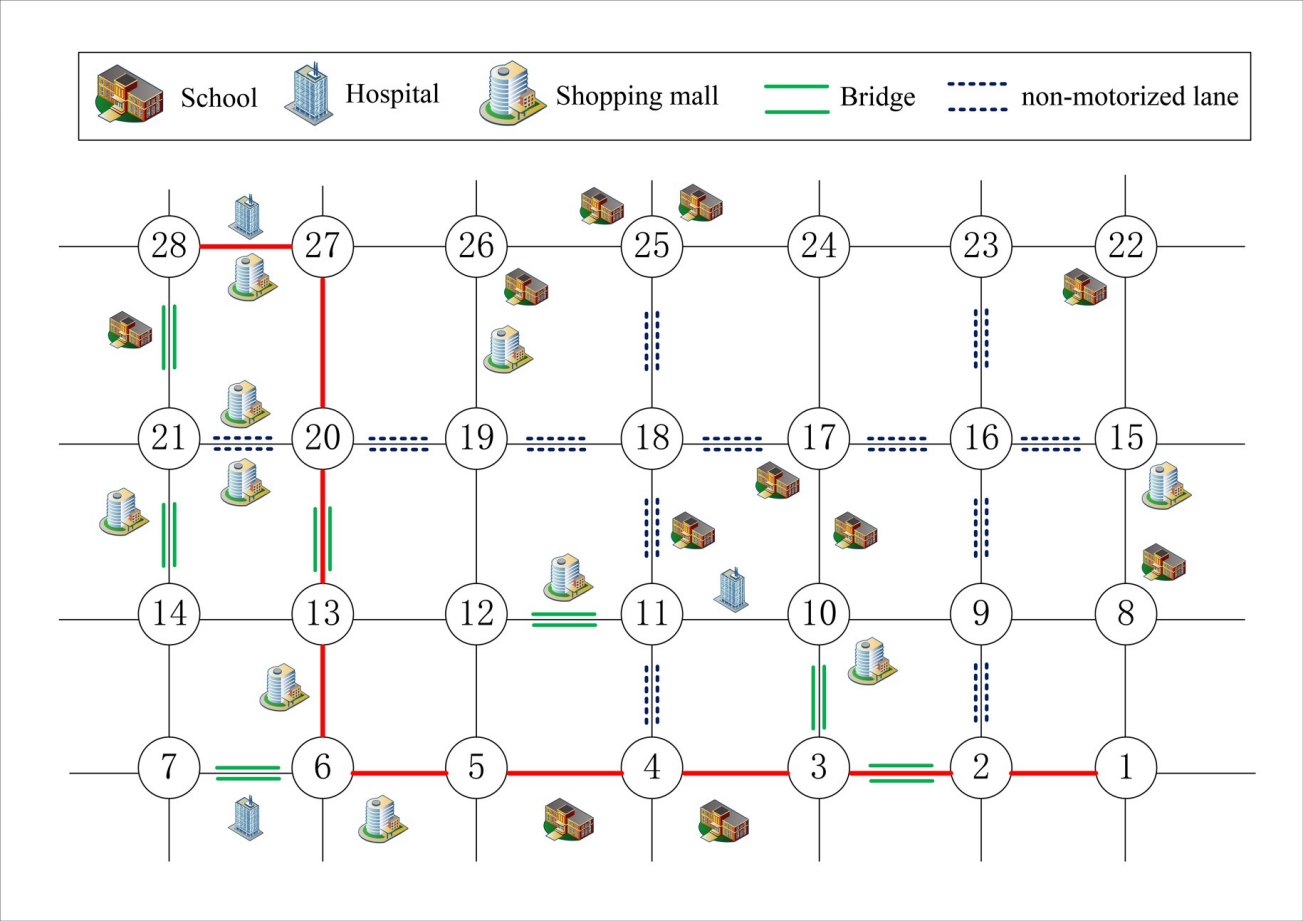
Time-consuming shortest route.

In order to verify the rationality of the model, in the case of *q* = 0.1, *a* = *b* = *c* = *d* = 0.25, the time-consuming preference coefficient *w*_1_ = 1, the number of initial population *n*_*pop*_ = 10, the individual length *l*_*pop*_ = 28, the maximum number of iterations *iter* = 200, and the probabilities of crossover and mutation are *pc* = 0.6 and *pm* = 0.005, respectively. The simulation results show that the comprehensive objective function value *F* = 100, the time-consuming function value *f*_1_ = 0.20, the risk function value *f*_2_ = 254.32. And the optimal route is 1-2-3-4-5-6-13-20-27-28, as shown in Fig 5. Baidu Maps Application, which is a free application with detailed street geographic information data and path selection function, is used for route comparison in the case. And the results show that two routes are consistent under the action of the shortest time-consuming target, indicating that the model is reasonable and can be used for further analysis.

### 4.3 Algorithm reliability analysis

**Fig 6:**
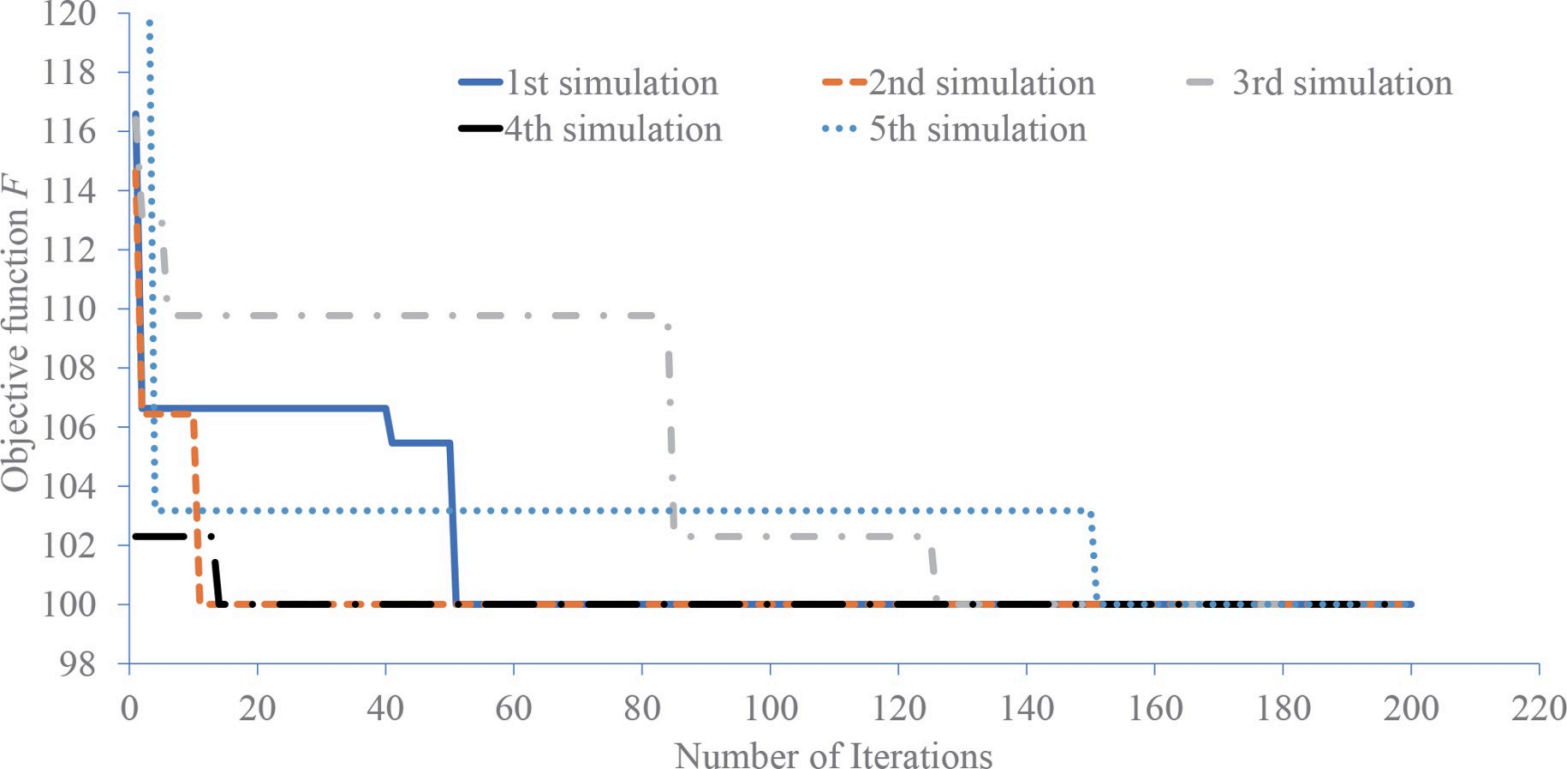
Comparison graph of objective function change.

The initial population in the model is generated by a random function. Under the working condition shown in 4.2, the model is used for five optimization experiments. The comparison of the change of the objective function is shown in Fig 6. It can be seen that the random population causes different initial objective function values, and also causes different routes and speeds of the objective function reaching the optimal value. However, the five experiments can reach the same optimal solution, indicating that the model solving algorithm will not fall into a local optimal solution due to the limitation of initial conditions. The model solving has no special requirements for the initial population, and has a wide range of applicability. Therefore, the design of the solving algorithm is reliable.

### 4.4 Comprehensive analysis

**Fig 7:**
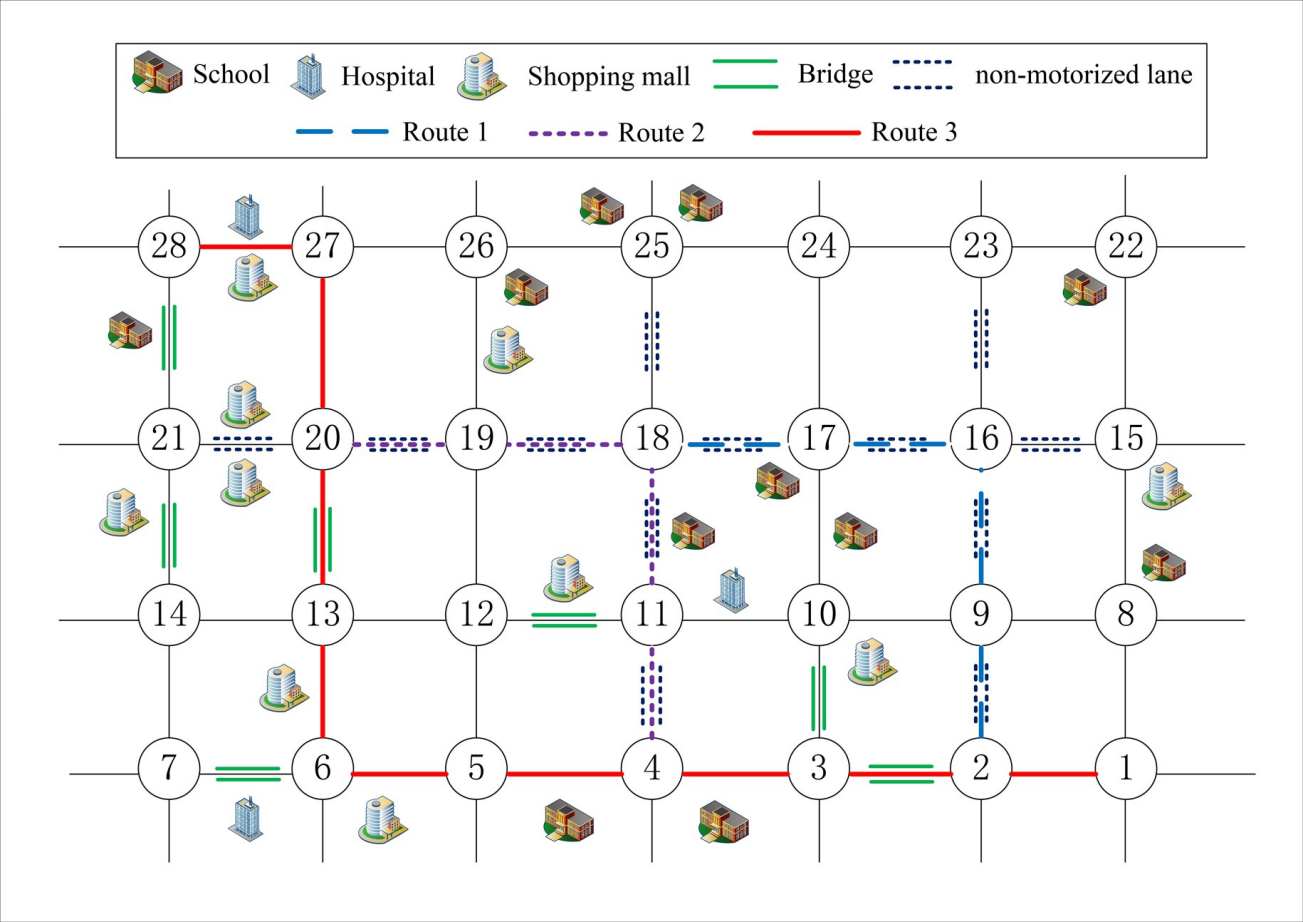
Route comparison chart.

By adjusting the time-consuming preference coefficient *w*_1_, the influence of user preference on the driving route is shown in Table 4 and Fig 7.

**Table 4:** Summary of routes comparison.

The value range of *w*_1_	Route	(*f*_1_, *f*_2_)	Time-consuming function parameters			Risk function parameters				
			Distance (km)	Number of traffic lights	Traffic flow complexity	Population (thousands)	Number of schools, hospitals, shopping malls	Number of bridges	Number of turns	Number of non-motorized road sections
[0.00,0.37]	1-2-9-16-17-18-19-20-27-28	(0.25,200.34)	8.11	20	Light congestion on the (27,28) section only	154.26	3	0	4	3
[0.37,0.66]	1-2-3-4-11-18-19-20-27-28	(0.21,215.10)	7.73	14		161.97	3	1	4	5
[0.66,1.00]	1-2-3-4-5-6-13-20-27-28	(0.20,254.32)	7.55	13		162.79	6	2	2	9

It can be seen from Table 4 that:

1. The model has the function of transportation vehicle path optimization. Given the user a time-consuming preference coefficient, a preferred route can be obtained. And through the simulation analysis, the preference coefficient changes in a certain range, corresponding to the same route, that path selection is not sensitive to the change of preference coefficient in a certain range. In the meantime, in dealing with multi-objective problems, different from the direct assignment of coefficient method, the model reveals the response relationship between user preference and path selection by adjusting the time-consuming preference coefficient, and replaces the fixed value with the interval range, which is more realistic [49]. In this case, when choosing the safest route, Route 1, the time-consuming preference coefficient range is the largest, about 0.37, while the minimum range is about 0.29 when Route 2 is chosen to compromise time-consuming and safety. Moreover, when Route 2 is selected, the interval ranges of time-consuming and safety preference coefficients are 0.37~0.66 and 0.34~0.63, respectively, indicating that when this route is selected, the coefficients of time-consuming and safety preference can reach 0.66 and 0.63 at the same time.
2. Comparing these three routes, Route 1 is the route with complete safety preference, Route 3 is the route with complete time-consuming preference, and Route 2 is the compromise route. It can be seen that compared with Route 3, although the driving distance of Route 1 increases, the number of risk sources such as population and bridges decreases, indicating that with the gradual decrease of time-consuming preference coefficient, the model has the effect of appropriately increasing time-consuming and reducing risk, which can help users choose safer routes. In the meantime, compared with previous studies that only considered population density as a risk source, this model considers important occasions such as schools and hospitals, which is more in line with public safety preference [40, 50]. Overall, compared with Route 1, Route 2 reduces time-consumption by 16% and increases risk by 7.4%, while compared with Route 3, it increases time-consumption by 5% and reduces risk by 15.4%. And it can be seen that route 2 risk reduction is greater than time-consuming increase. Moreover, the corresponding coefficients of time-consuming and safety preference can reach about 0.65, and the relevant stakeholders have high acceptance of the route. Therefore, Route 2 is the optimal transportation route under the case conditions.
3. For different research areas and working conditions, the range of preference coefficient corresponding to the optimal route is different. Transportation enterprises need to comprehensively consider the acceptability of preference coefficient by enterprises themselves and the public. In the meantime, they also should measure the acceptance of transportation cost changes caused by route changes on transportation efficiency, fuel consumption and risk management, so as to determine the feasible optimal route under different working conditions.

## 5. Suggestions

**Fig 8:**
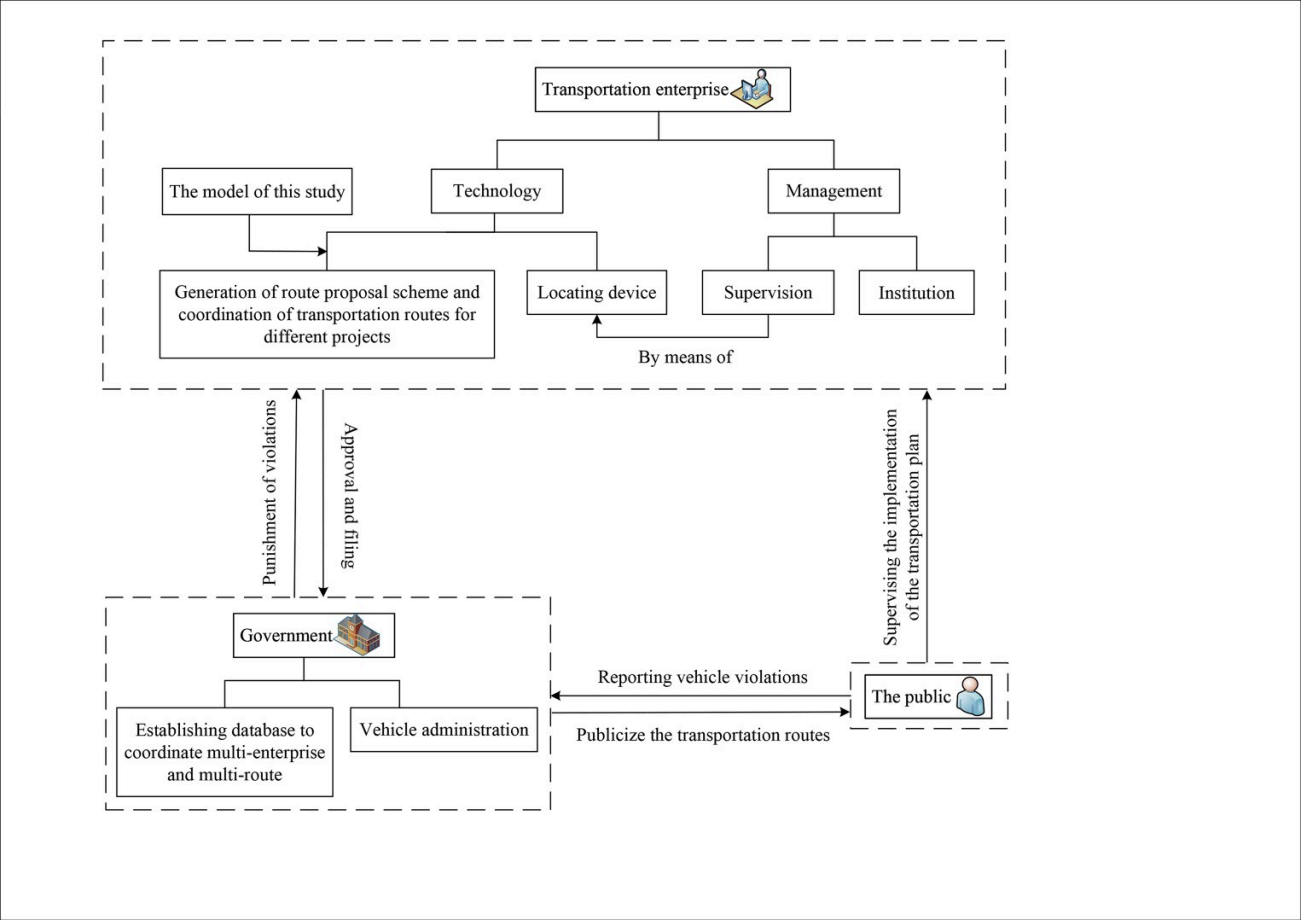
Management mechanism of transportation route.

This study proposes a path selection method to balance the time-consuming and safety of construction vehicle transportation, which makes it possible to effectively improve the management level of construction vehicles by establishing the urban transportation route management mechanism of construction vehicles as shown in Fig 8. In terms of the construction of this mechanism, the following suggestions are put forward:

1. Construction of the government through the system to improve the leading, transportation enterprises in-depth implementation, the public and law enforcement unit effective supervision of construction vehicles transportation route management mechanism.
2. Improve path optimization method and process from the system. Firstly, the enterprise demonstrates the routes and puts forward the transportation scheme. And then the transportation routes are publicized and accept the supervision of the community after approval of the relevant administrative departments.
3. Transportation enterprises should strengthen the construction of route management system, improve the understanding of route management, and strengthen self-management by real-time positioning monitoring, staff skills training, regular vehicle maintenance.

## 6. Conclusions

Aiming at the difficulty of urban driving risk management of construction vehicles, a time-consuming-risk bi-objective path optimization model is established. The conclusions are as follows:

1. The model is proved to be reliable in path selection and has the function of balancing time-consuming and safety. In this case, the optimal route is more than the single-objective optimal value to increase time / risk, and the game obtains 2~3 times of security / economic benefits.
2. The coefficients of time-consuming and safety preference can reach a high level at the same time, which has positive effects on the promotion of the route. And in this case, the coefficient is about 0.65.

The research results are affected by the selection and value of model parameters, case characteristics and other factors. It is necessary to continuously explore more refined path selection methods in different types and more complex conditions. In the meantime, this model still has the problem of insufficient collection of road risk sources, and lacks a more detailed distinction between the spatial and temporal changes of road environment. Finally, different from the traditional one-point to multi-point distribution scheme, the model is only applicable to the single point to single point transportation route selection problem, focusing on the consideration of urban transportation characteristics.

## Supporting information

S1 File (TXT)

S1 Dataset Case information. (XLSX)
